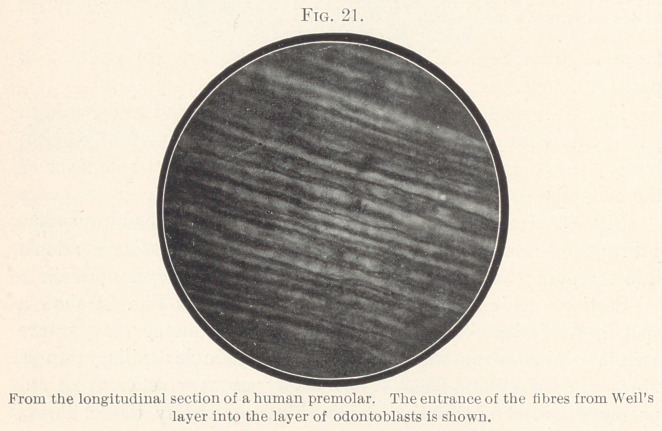# Some Histological Facts That Contradict the Generally Accepted Odontoblast Theory

**Published:** 1904-03

**Authors:** Michael Morgenstern

**Affiliations:** Strassburg, Germany


### International Dental Journal.

Vol. XXV. March, 1904. No. 3.

### Original Communications.[1]

### SOME HISTOLOGICAL FACTS THAT CONTRADICT THE GENERALLY ACCEPTED ODONTOBLAST THEORY.[2]

BY MICHAEL MORGENSTERN, STRASSBURG, GERMANY.

Mr. Chairman and Members of the Section,—The committee of the Section on Stomatology of the American Medical Association has been kind enough to invite me to address you on the subject of my investigations on the pulp. While I feel very much honored by your kind invitation, I nevertheless approach my task with some trepidation, because my histological examinations lead me to conclusions that are considerably at variance with those that others who have worked along similar lines have arrived at in the course of the past years. It is this fact that makes it somewhat embarrassing for me to address you. In Germany I have for the present only a small number of followers, and I fear that the majority of you who are assembled here to-day are also fanatic

[1] The editor and publishers are not responsible for the views of authors of papers published in this department, nor for any claim to novelty, or otherwise, that may be made by them. No papers will be received for this department that have appeared in any other journal published in the country.

[2] Lecture with lantern-slide demonstration, read before the American Medical Association, Section on Stomatology, at New Orleans, May 5 to 8, 1903.

adherents of the old odontoblast theory; consequently I assure you that I expect only a moderate amount of applause and of approbation at your hands.

But it is in your country precisely that the first voices were raised years ago against the prevailing views in regard to the rôle of the odontoblasts, and while I do not fully share the views of Heitzmann, Bödecker, Abbott, and Andrews, I nevertheless recognize in these men brave pioneers, and their example inspires me with the courage to appear with my results before an audience that is composed of the greatest investigators in our particular field of research.

Fig. 1.

Multiple layer of odontoblasts at the tip of the dentine germ from the cuspid tooth of a new-born infant. Longitudinal section.

According to the views that are prevalent in regard to the rôle of the odontoblasts, the surface of the pulp is covered with a single layer of cells that are in intimate contact with each other like epithelium; these are the dentine cells, or odontoblasts, each one of which sends a protoplasmic process (dentine fibre) into the dentine. It is claimed that these odontoblasts are perennial,—*i.e.,* that they persist during the formation of the dentine, and are neither reduced in number during this process nor replaced by other cells. It is further claimed that the nutrition and the sensation of the dentine is exclusively bound to these dentine cells and their protoplasmic processes. All subsequent formative changes in the dentine, as, for instance, the genesis of the transparent zone in caries and the broadening of Neumann's sheath in senile teeth, are also believed to be exclusively due to metaplastic activity going on in the protoplasmic processes of the odontoblasts.

The great advantage of this theory is its simplicity, for the most complicated physiological processes can be explained on a very simple, one might almost say, unitarian histologic basis. The very fact, however, that everything seems so simple should lead us to be sceptical. Recent investigations into the structure and the composition of the albumins teach us that things that appear exceedingly simple are nevertheless most complicated, and it seems hardly prob-

Fig. 2.

Multiple odontoblast layer in process of degeneration, from the pulp of an incisor tooth of a new-born calf. Longitudinal section.

able that different physiological processes can be explained on the basis of one histologic unit. True, the adherents of the odontoblast theory point out that in the lower orders of animal creation a single cell or group of cells may occasionally assume all the functions of the organism and that similar conditions might obtain in the case of the odontoblasts; against this argument we can formulate the objection that the odontoblasts are simply parts of a more complicated organism, and that in higher orders of animal creation the anatomic differentiation proceeds *pari passu* with the physiologic division of labor that becomes operative in complicated organisms; one might further object that in no vertebrate is any other cell variety known to exist whose protoplasm can at the same time perform the functions of nutrition, of sensation, and of metaplastic transformation.

These theoretic objections alone do not, however, lead me to doubt the correctness and the validity of the odontoblast theory as it is accepted to-day; I have, moreover, been able to discover a number of histological facts that cannot be reconciled with this

Fig. 3.

Total degeneration of the multiple odontoblast layer at the tip of the dentine germ of a temporary molar tooth of a new-born infant. Longitudinal section.

theory. It is the main object of this address to demonstrate these histological facts to you with the aid of lantern-slides that I have made from my microscopic preparations.

1. I have frequently seen, in the so-called pulp-horns of the dentine germ in the fœtus and the new-born, the dentine cells arranged in a multiple layer. Fig. 1 is one of a series of sagittal sections through the anterior dental germs of a new-born child. You will notice that each dentine cell is pear-shaped; you will also notice the direction of each dentine fibre and of the capillaries, for all these features show that we are actually dealing with a longitudinal section and not with a diagonal section in which the multiple arangement of the cells might be simulated. The layer I am discussing consists of ten to twenty rows of cells. The individual cells show no trace of lateral flattening from mutual pressure; on the contrary, capillaries and fibrillæ will be seen running between the different cells in a direction that is parallel to the longitudinal

Fig. 4.

Changes in the odontoblasts before the beginning of dentinogenous disintegration. From the tip of the dentine germ of an incisor tooth of a sheep's fœtus.

axis of the dentine germ. It is clear, therefore, that the cells are not arranged in several layers on account of crowding or lack of room and mutual pressure.

The structural changes in the dentine cells also call for discussion. It will be seen that the cells become paler from the bottom towards the surface of the layer, that the nuclei disappear, and that the cell boundaries become indistinct, until finally near the margin of the dentine hardly any cell remnants can be seen; for in this zone the changes in the substance and the structure of the cells are so far advanced that the dentine cells have become fully disintegrated and have disappeared from view.

FIG. 5.

From the longitudinal section of a portion of the pulp from a normal premolar tooth of a girl of twelve years. Multiple layer of odontoblasts and demonstration of all the vessels and nerves of the pulp, particularly of the spindle-shaped cells of Ranvier.

If we compare Fig. 2, made from a frontal section through the pulp-ridge of a young tooth, with Fig. 1, we will find the same

FIG. 6.

Last stage of conjugation of dentine cells, from the tip of a molar tooth of a young rabbit, the tip having undergone partial calcification.

conditions. Here again we see a broad layer of oval and round cells, that are already beginning to become pale, extending up to the edge of the dentine; the layer here consists of some twelve rows of cells. These cells are odontoblasts in a state of dentinogenous metamorphosis, and show evidence of beginning disintegration. Between the cells we see capillaries running in the same direction as the dentine canaliculi. Below this zone we see the pulp-cells in close aggregation, arranged in rows and extending their processes into the layer of disintegrating odontoblasts; these cells are in a state of conjugation or of disintegration. These processes lead to the formation of odontoblasts.

Fig. 7.

First stage of conjugation of the mesodermic cells of the dentine germ for the formation of odontoblasts; also some preformed dentine fibres in places where no odontoblasts as yet exist. From the dentine germ of a human fœtus.

In preparing a tooth-germ according to the method of Koch-Weil, and in making a section without decalcification, spherical solid structures are seen in place of the pale disintegrated odontoblasts; these spheres were formerly considered to be dentine spheres or globular masses, and were described by these names. In another stage of the formation of dentine a transparent homogeneous layer composed of single trabeculæ is occasionally seen in place of the disintegrating odontoblasts.

If a tooth is still in a developmental stage, and is only incompletely decalcified, spherical structures are also seen in the place of disintegrating odontoblasts; many of these little globules, however, contain remnants of cells and nuclei; in injected specimens

I was even able to detect capillaries between the spheres. These capillaries could be sharply differentiated from the colorless spheres of dentinogen by their intense color.

After this discursion you will be able to understand Fig. 3, prepared from a frontal longitudinal section through the germ of a molar tooth in a new-born infant. The preparation was decalcified until all the calcium salts were removed. In the natural state dentinogenous masses of spheres were found throughout the whole visible portion of the pulp; after careful and thorough decalcification a wide layer of disintegrating cells was seen instead, in which

Fig. 8.

First formation of dentine and trabeculæ of odontoblasts without nuclei. From a longitudinal section of the dentine germ of a cow's tooth.

odontoblasts could no longer be found. Towards the centre, pulp-cells are seen that are odontoblasts and that send processes or fibrillæ into certain portions of the area of disintegrating cells and through this zone into the dentine.

In a later stage of formation dentine appears in the place of the mass of globules and the pulp-cells are seen to have undergone metamorphosis into trabeculæ of odontoblasts; some of them become distended and appear as cartilage cells that are surrounded by a light areola. This is the stage that preceded the disintegration or the dentinogenous formation of globules and that is illustrated in Fig. 4. This specimen is prepared from a longitudinal section through the dentine germ of a sheep's fœtus.

As long as the formation of dentine continues, the arrangement of dentine cells in multiple layers can be demonstrated. In Fig. 5, made from the longitudinal section through a normal bicuspid in a girl of twelve years, the layer of odontoblasts still consists of seven to eight rows of cells. I have been able to show that innumerable rows underneath each other are merely different layers of a single row that have become displaced so as to lie below one another, but really consist of several rows of cells. In Fig. 6, representing the partially calcified pulp-horn from the back tooth

Fig. 9.

Narrow layer of dentine, with inclosed odontoblasts, from the root of a young cow's tooth. Transverse sections. Total metaplastic transformation of some of the odontoblasts of the odontoblast layer.

of a rabbit, you can see, particularly in the centre of the slide, how the arrangement in rows develops and how a single fibrillar process starts from several odontoblasts. The light spots between the cells are young dentine, so-called secondary dentine.

I have been able to demonstrate this conjunction of pulp-cells for the purpose of odontoblast formation in the earliest developmental stages. In Fig. 7, made from the tooth-germ of a human fœtus, the genesis of the small number of odontoblasts that is formed in this specimen from confluence of conjugation of pulpcell (mesoblasts) can be clearly seen. In those places in which no odontoblasts have been formed, single fibrillæ—*i.e.*, preformed den-

Fig. 10.

Formation of dentine in the root of a cow's tooth without typical odontoblast layer; stage of conjugation of dentine cells in the dentine and in the pulp; direct transformation of dentine cells into dentine substance.

tine fibres—appear underneath the layer of enamel cells at the margin of the dentine germ.

Fig. 11.

First formation of dentine from the root of a young cow's tooth. Gradual transformation of pulp-cells to odontoblasts and of those to dentinogenous globular masses and trabeculæ. Typical layer of odontoblasts not present.

In a somewhat later stage and after dentine has already been formed, odontoblasts arranged in trabeculæ appear; many of these later odontoblasts, however (as shown in Fig. 8), no longer possess a nucleus, whereas the odontoblasts in Fig. 7 may even possess two or three nuclei.

I believe that I have adduced valid arguments against the existing odontoblast theory by the demonstration of Figs. 1 to 8, for I have been able to show (1) that, at least in the earliest developmental stages of the teeth, an odontoblast originates from several cell units, (2) that during the different periods of tooth formation a multiple layer of odontoblast cells can be demonstrated, (3) that, finally, the external rows of this layer undergo metaplastic transformation, so that they disintegrate; consequently I argue that it is impossible for each odontoblast to persist throughout the whole period of tooth formation without being destroyed and subsequently replaced by other cells from which the dentine is ultimately formed.

Fig. 12.

From the same specimen as Fig. 11, higher power. The first formation of a typical layer of odontoblasts disappears at once, owing to the rapid transformation of the latter into the dentine; here those dentine cells that are still in a stage of metaplastic transformation appear enclosed in finished dentine.

The innumerable smaller cells that are seen underneath the layer of odontoblasts have been completely ignored by the adherents of the odontoblast theory. The fact, moreover, that the nuclei of the odontoblasts show no karyokynetic figures has been misinterpreted by them. The replacement of the odontoblasts that are destroyed in process of dentine formation does not occur by cell regeneration and karyokynesis of nuclei, but by proliferation of the small underlying pulp-cells that are connected by innumerable protoplasmic anastomoses with the dentine cells.

2. The salient feature of this whole investigation is to show that dentine cells are actualy used up in the process of dentine formation. If it should be possible to demonstrate the presence of transition forms between the different forms of cells that are in process of metaplastic metamorphosis this postulate would be fulfilled. When the formation of dentine proceeds quietly and uniformly, such transition forms are not easy to find; when the formation of dentine, however, proceeds in an irregular manner,—*i.e.*, when "functional irritants influence the formation of dentine,"—

Fig. 13.

A somewhat broader layer of dentine, with inclosed dentine cells that are partially arranged in rows or groups. Transverse section through the root of a young rabbit.

then it is an easy matter to find such forms of cells. I take the liberty of demonstrating by the following slides what I wish to show; the specimens are taken from the roots of teeth from ruminants, rodents, and fish. I have already, in a separate monograph, described the results of these particular investigations on the formation of dentine in these species.

Figs. 9 and 10 are made from cross-sections through the root of a cow's tooth in its first stage of development. We see a narrow strip of dentine with fenestrations; within the openings of the dentine are seen odontoblasts, some of them still connected with one another by protoplasmic processes; the substance of the majority of the cells is changed, and, owing to "dentinogenous transformation" that is taking place, they appear black. In the narrow odontoblast layer we see pale odontoblasts, and, in addition, some cells that are colored black; in this layer, therefore, we see the same change in the substance of the cells throughout.

In Fig. 10 the layer of odontoblasts is irregular, somewhat

Fig. 14.

Plugs of pulp remaining in young dentine. They consist of columns of dentine cells that have undergone partial metaplastic change; consisting also in part of small pulp-cells and capillaries.

broader than above, and interrupted in many places by protruding plugs of dentine. The odontoblasts of this layer correspond, in regard to arangement, form, and substance, to those that are found in the interior of the dentine.

The gradual transition of pulp-cells into the layer of odontoblasts, as well as the transition of the odontoblasts into particles of dentine, can be seen clearly in Figs. 11 and 12; these sections are made from the first dentine appearing in the roots of a cow's tooth. In Fig. 11 the changes in form that the odontoblasts undergo in the process of dentine formation can be seen particularly

Fig. 15.

Plug of pulp in the dentine in which the nuclei still appear quite unchanged. From the tooth of a sheep's fœtus.

well, and in Fig. 12 the arrangement of the pulp-cells and their gradual transition to odontoblasts is especially clear.

Fig. 16.

Different cell territories in the dentine of a molar tooth of a human premolar. Fibres passing from layer of odontoblasts deep in pulp.

In Fig. 13, in which we see that the dentine is broader, the arrangement in rows, the conjugation, and the changes in the substance of the odontoblasts can be seen on a somewhat larger scale.

In Fig. 14 the dentine contains two plugs consisting of different kinds of cells; the other margin of these plugs is very pale, and is seen to be undergoing metamorphosis to dentine.

In Fig. 15 a plug of cells within the dentine is shown; here we see the nuclei unchanged, while the protoplasm is beginning to pale. This condition is always seen immediately before the dentinogenous metamorphosis begins.

These plugs that frequently pass through the layer of odontoblasts and almost penetrate to the cement layer in the dentine may easily create the impression that dentine had been previously formed, had again been absorbed, and had been subsequently re-

Fig. 17.

A few perennial cells from the dentine of a molar tooth of a somewhat older rabbit.

placed by pulp-tissue that had penetrated into the spaces created in this way. This view, however, is opposed by very valid arguments. I have studied the peculiar formation under discussion through all developmental stages,—*i.e.,* from the very beginning of tooth formation to the completion of root growth,—and I could determine on the basis of thousands of observations that these plugs never penetrate into finished dentine, but are always found before the formation of dentine has begun. The plugs represent masses of pulp that remain behind in certain areas owing to an irregular development of the dentine; later these plugs are destined to undergo the same metamorphosis as other portions of the pulp that are further advanced in their development. Finally these plugs disappear and the area they occupy is replaced by normal dentine.

Gerö Rudas has recently stated that the presence of cells in the dentine is always due to the invasion of leucocytes, and that the development of the pulp is deficient in those places in which incapsulated pulp-cells are found; I am forced, however, to deny the correctness of these statements from my own experience. I believe I have shown that the cells within the dentine do not consist of invading pathologic leucocytes, but of mesodermic cells and dentine cells that have been passively inclosed. Under normal conditions these cells disappear without leaving a trace; only in those cases in which the developmental processes are arrested—as, for instance, by rapid and great destruction of the cutting edges of the teeth from overuse, with resulting atrophy of the pulp—do we see deficient development of dentine in such places.

In the dentine of certain animals, however, we sometimes see single cells, cell groups, and cell territories that probably persist during the life of the animal; they must either persist as living cells that are normal or as decayed cell rudiments. I have been able to find such cells particularly in the teeth of rodents, and most frequently in the molar teeth of rabbits. In Fig 16 such cell territories are shown, and in Fig. 17 single cells inclosed in the dentine of rabbits. We know nothing positive in regard to the significance of those cells.

What we learn from the formation of dentine under the influence of functional irritants is that dentine may be formed without a typic layer of odontoblasts, that the pulp-cells that were heretofore considered to be completely indifferent are directly concerned in this process, and, finally, that the latter are arranged in rows during this developmental process without ever being transformed into typic odontoblasts. The transformation of these cells into dentine may, however, be followed step by step, for it can readily be shown that they as well as the odontoblasts that are enclosed within the dentine are gradually converted into dentine; and this, I argue, proves that in the formation of dentine, dentine cells are used up.

*At all events, we may assume positively that no conclusions can be drawn from the fact that the odontoblasts form one layer. In regard to the permanence of the odontoblasts, nor in regard to the continuous disintegration of these cells and the replacement of*

*the destroyed cells by new ones, I am inclined to the belief that the formation of a single layer of odontoblasts constitutes merely a transitory stage in the development of dentine, and a stage, moreover, that need not necessarily be observed in all cases.*

3. In connection with the old odontoblast theory, the idea is often expressed that the perennial dentine cells (whose number always remains the same) advanced towards the pulp as well as towards the dentine, and that in process of advancing towards the pulp they displace this tissue; the question is not decided, however, whether or not all the constituents of this displaced pulp undergo atrophy or whether they are merely compressed. In the light of my discovery of the metamorphosis of pulp-cells into dentine cells and of the continuous degeneration of the latter this view is altogether untenable.

Only one explanation seems satisfactory,—namely, that more and more of the pulp becomes included in the process of dentine formation. We must assume that the pulp becomes smaller and smaller, and we do not need to postulate compression nor atrophy of the pulp to explain its gradual shrinkage.

What becomes of the blood-vessels and the nerves in those portions of the pulp that are converted into territories of dentine cells?

I attempted to solve this query many years ago and have made it the subject of a comprehensive investigation. The main results of my studies have been described in a lecture. I found that the blood-vessels undergo atrophy or are converted into intraglobular spaces. I also showed that the nerves, with exception of the axis cylinders, are converted into a hyaline substance that later undergoes calcification, and that the axis cylinders partially atrophy and partially persist. At the same time I demonstrated that these cylinders are very difficult to demonstrate histologically.

One of my arguments in favor of the view that new pulp areas are constantly included in dentine formation is that the histologic structures within and below the odontoblast layer undergo continuous changes. At one time we may see numerous capillaries between the odontoblasts, occasionally even between them and the layer of dentine; in other cases a layer of odontoblasts will be found containing no trace of blood-vessels. Sometimes the pulp immediately below the odontoblasts contains very many cells, and sometimes it contains very few. Frequently a large number of very fine fibres are seen in this zone, constituting what is called Weil's layer; in other instances this layer will be found to consist of a finely granular material containing neither cells nor fibres. The vaso-dentine of fish teeth is a classical example of the continuous participation of pulp territories in dentine formation without the agency of odontoblasts.

If we look at the question in this light, the dentine fibres must need assume an altogether different histogenetic relation to the odontoblasts than has hitherto been postulated; it seems impossible that the fibres should arise exclusively from the odontoblasts that are situated at the surface of the pulp, and they must also be im-

Fig. 18.

Between the dentine and the odontoblasts is seen a finely granular zone from which the dentine cells have already disappeared; this zone is traversed by numerous preformed dentine fibres. Tooth from a young rabbit.

agined to arise in part from pulp-cells situated deeper down in the pulp; in other words they must be more or less preformed in the pulp and cannot be said to originate by metaplastic transformation as a residuum of the dentine cells in the process of dentine formation.

The next figures demonstrate these postulated conditions. In Fig. 18, made from the molar tooth of a young rabbit, a layer is seen underneath the young dentine that contains very few cells and that is traversed by fibres. Here dentinogenous disintegration of odontoblasts has taken place and the dentine fibres originate from the deeper cells.

In Fig. 19 made from a longitudinal section through the dentine germ in a sheep's fœtus, numerous fibres will be recognized that pass from the dentine through the multiple layer of odontoblasts, and that in part originate below this layer. I observed that many of these fibres belong to the nervous system, and originate, as will

Fig. 19.

The layer of odontoblasts is traversed by numerous dark fibres that in a great part originate externally to it and that pass into the dentine. Longitudinal section through the dentine germ of a sheep's fœtus.

be seen, from nerve-branches in the pulp that are running a parietal course.

Fig. 20 is made from the same series of cuts, and under a high power shows fibres that run between the multiple layer of odontoblasts; in other words, these fibres pass through the whole layer of the odontoblast cells into the dentine.

Fig. 21 is made from the pulp of a young human premolar. Fibres will be seen passing from the layer of odontoblasts deep down into the pulp; these fibres constitute the so-called layer of Weil.

Sections made from the same series of cuts as Fig. 21 show a spot in Weil's layer under a higher power. Numerous fibrillæ are seen to originate from the circumference of a single capillary; these fibres enter the odontoblast layer. They are very different in appearance; most of them stain like fine connective tissue fibres; some of them are fine tubules that are occasionally found to contain a few red blood-corpuscles; in most cases, however, they are so fine that blood-corpuscles cannot pass through. Formerly I described these tubes as lymphatic channels, but am now inclined

Fig. 20.

The same as Fig. 19 under a higher power.

to the belief that they are blood-vessels and that they constitute very fine nutritional or tissue fluid fibres of a variety that has

Fig. 21.

From the longitudinal section of a human premolar. The entrance of the fibres from Weil's layer into the layer of odontoblasts is shown.

hitherto never been described in dental tissues. By a special method of preparation I have been enabled to demonstrate large numbers of them between the dentine cells, and could also repeatedly demonstrate their presence in dentine.

A large number of these fine channels is shown between the odontoblasts in a molar tooth of a young rabbit. Some of these odontoblasts are in process of dentinogenous disintegration, and in most of them only the nuclei are preserved. These channels, that vary in thickness from one-fourth to one-third the width of a cell nucleus, usually terminate in a knob-like loop.

A very thin capillary in the layer of odontoblasts and another one in a cell group situated in the dentine of a young calf are shown.

I call your attention particularly to these channels and capillaries that pass between the dentine cells, for there is resemblance in regard to distribution and location between these structures and the dentine fibres; in Figs. 20 and 21, both structures are seen to pass side by side through the odontoblast layer and below it, —*i.e.*, in Weil's layer. But I have also been able to show repeatedly that in addition to Tomes's fibres fine channels frequently enter the dentine, the method I employed for demonstrating their presence being either injection of the specimens or special preparation of a series of cuts made from the fœtus of cows, fish, and sheep.

4. The question of the nutrition of dentine can only be solved after the relation existing between Tomes's fibres and the fine channels that I have demonstrated between the odontoblasts is explained.

I believe, however, that I have definitely solved the question of the sensibility of dentine by my demonstration of the entrance of nerves into the dentine. That the sensibility of dentine is not dependent on the presence of odontoblasts in the dentine, as taught by Black, Walkhoff, and other authors, could be demonstrated clinically by the fact that teeth whose pulps were completely decalcified and that contained no more odontoblasts were still highly sensitive. In these cases of general calcification the axis cylinders were not involved in the process and remained intact.

Boll in 1868, Bödecker in 1883, I in 1892, and Romer in 1899 demonstrated the fact that pulp-nerves pass into the layer of odontoblasts. The deviation of nerve-fibres between the odontoblasts and the dentine was observed in 1892 by Retzius, and by Huber in 1899; the entrance of nerves into the dentine I demonstrated in 1892, and Romer in 1899.

In older specimens nothing but pale fibres are seen in those places where nerves enter into the dentine; these fibres, however, are clearly differentiated from the other tissues; this may be seen in an old specimen prepared from a young rabbit's tooth.

In preparations made according to my modification of Mallory's stain, the axis cylinder is clearly different from all other stained tissues; preparations of this character, as will be seen from the following figures, can be preserved for a very long time.

In a specimen made from the dentine germ of a sheep's fœtus a few of the axis cylinders that run towards the centre of the odontoblast layer between the dentine cells can be clearly recognized; they pass from the parietal sheath of the nerves of the dentine germ, and can be followed through the whole odontoblast layer beyond the boundaries of the dentine.

In one specimen the entrance of nerves into the dentine can be seen still more clearly; one of the axis cylinders is seen to terminate in a knob-like ending in the dentine, and another one to terminate in a similar way before reaching the dentine—*i.e.*, in the odontoblast layer.

The course of the nerves in the odontoblast layer varies greatly.

They often traverse the layer in the same direction as the dentine channels and the odontoblasts; in some places they deviate from this direction. Sometimes they form a veritable plexus with their small lateral branches; such a plexus in the odontoblast layer can be seen in specimen. The nerve fibrils *apparently* originate from cells and cell nuclei; if one looks carefully, it will be seen, however, that they are merely close to the cells and really pass alongside of them.

At the point of division of the nerves of the odontoblast layer I have frequently seen one or more spindle-shaped enlargement.

In conclusion, I take the liberty of demonstrating the course of the nerve-fibres within the dentine channels in a cow's tooth. The preparation was made according to Golgi by the sublimate method after the specimen had previously been injected with methylene blue. The fibres of Tomes appear pale; the fibres that I call axis cylinders are darker and frequently form spirals around the dentine fibres; they are situated between the walls of the dentine channels and the fibres of Tomes.

Basing on a series of histologic facts that I have attempted to illustrate to you with the aid of a few photographic slides of some of my specimens, I believe I have demonstrated that the generally accepted odontoblast theory, the theory of dentine formation, and of the sensibility of the dentine are untenable. While I do not dare to substitute a new and complete theory for the old one, I hope, nevertheless, to have contributed a few new facts by my investigations that may form the basis of an odontoblast theory that will satisfy us both in an anatomic and physiologic sense.

## Figures and Tables

**Fig. 1. f1:**
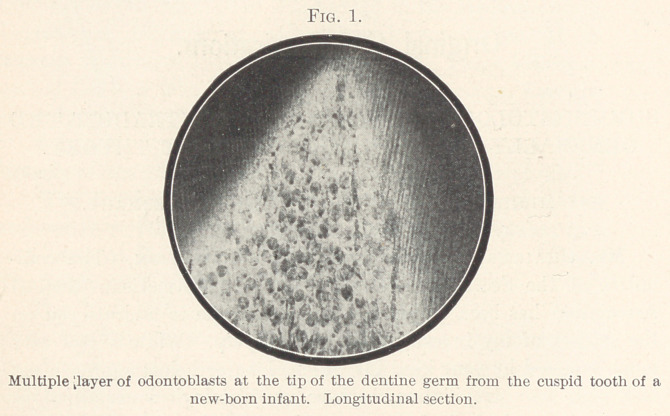


**Fig. 2. f2:**
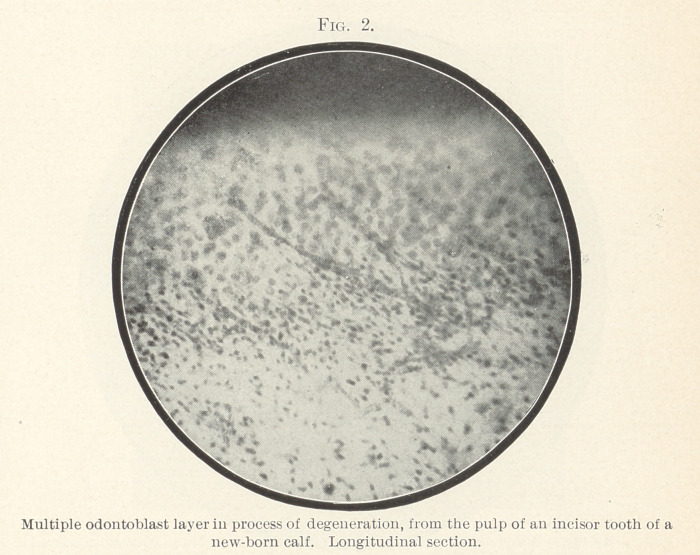


**Fig. 3. f3:**
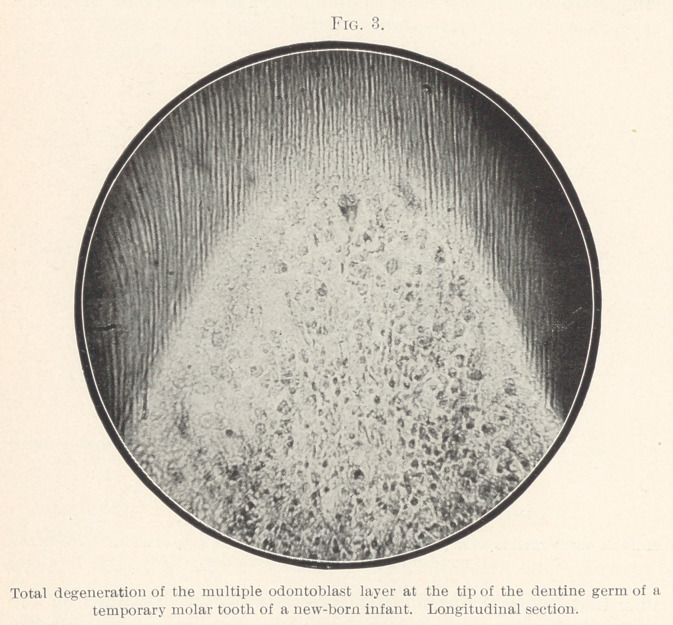


**Fig. 4. f4:**
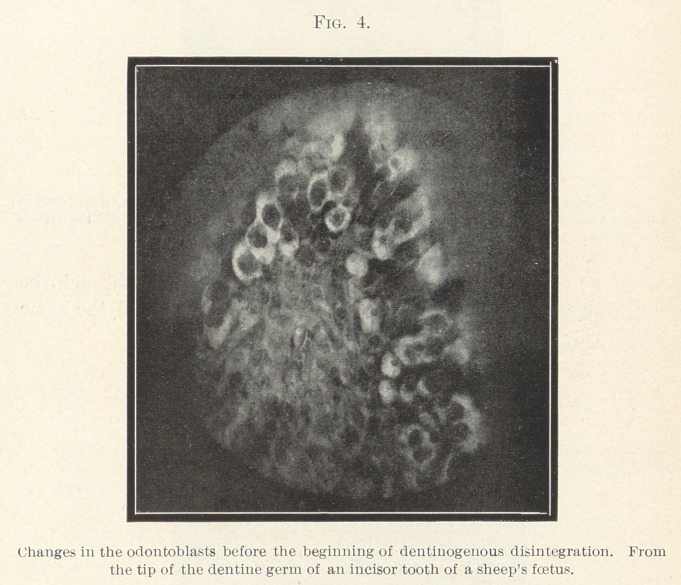


**Fig. 5. f5:**
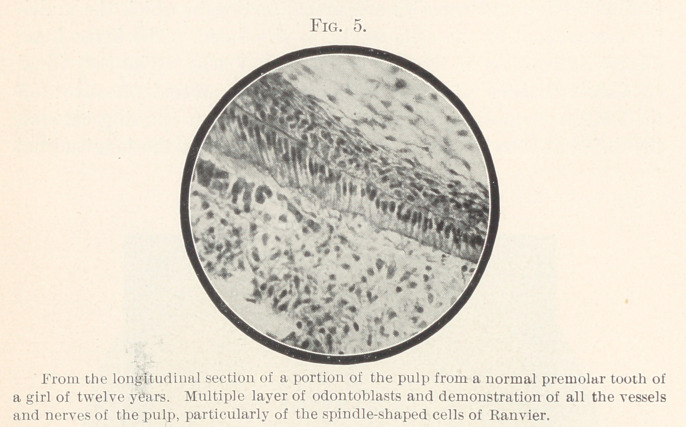


**Fig. 6. f6:**
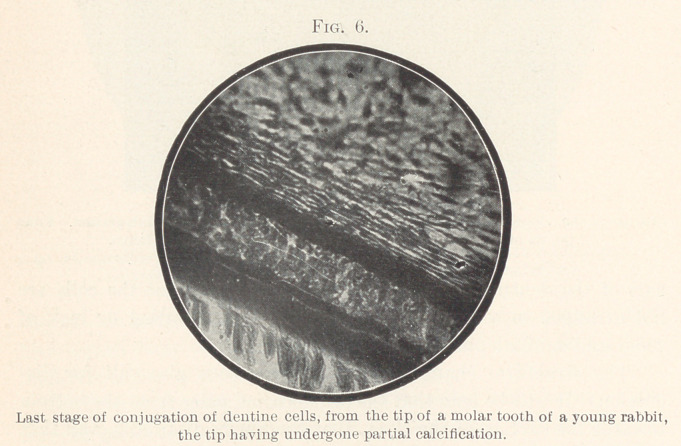


**Fig. 7. f7:**
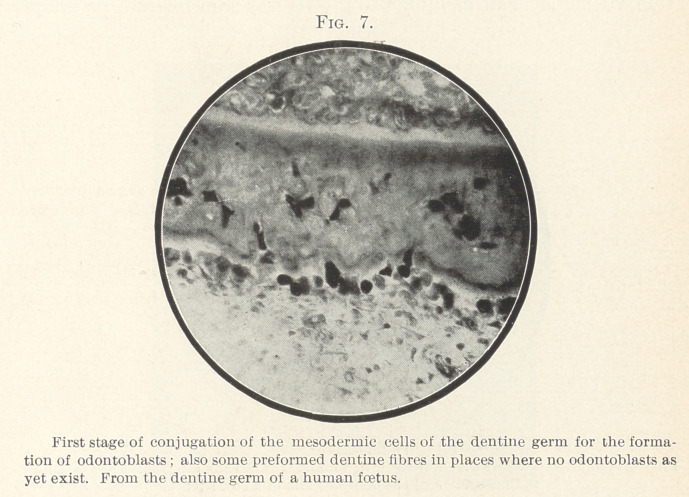


**Fig. 8. f8:**
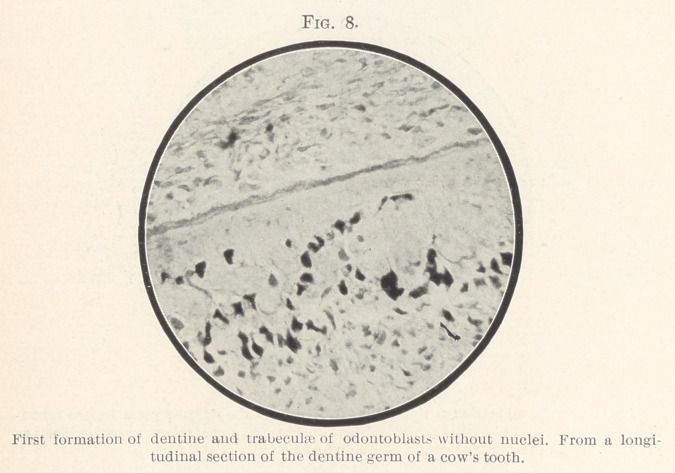


**Fig. 9. f9:**
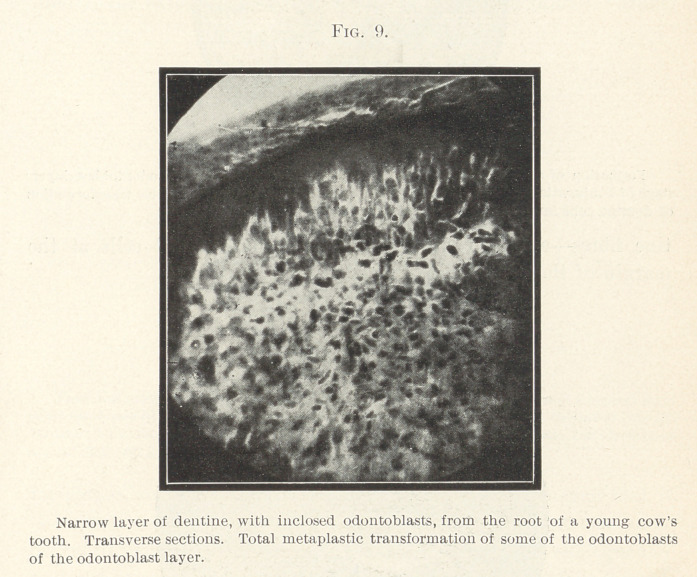


**Fig. 10. f10:**
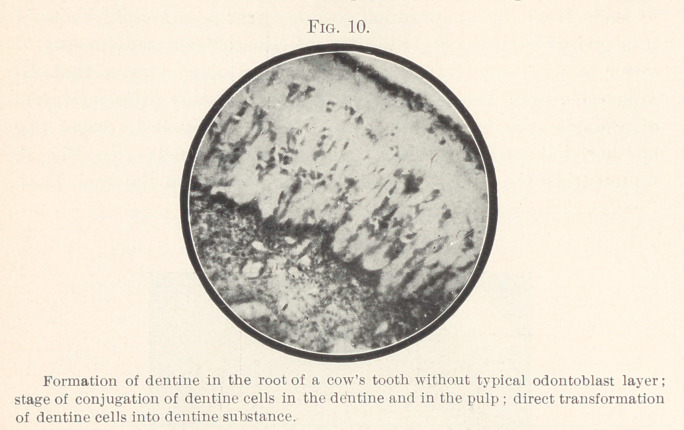


**Fig. 11. f11:**
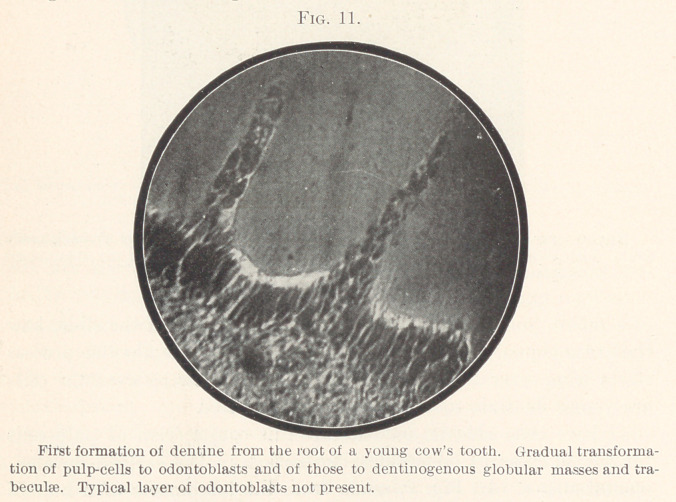


**Fig. 12. f12:**
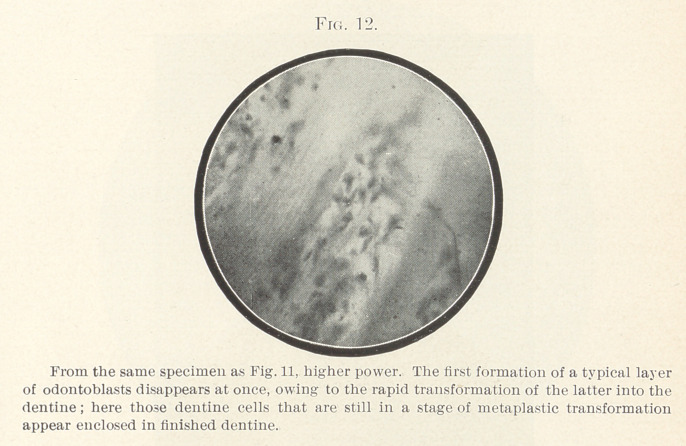


**Fig. 13. f13:**
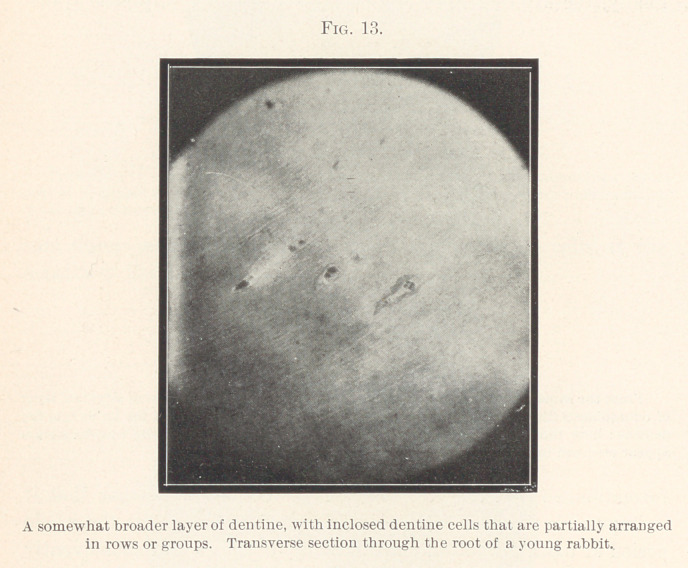


**Fig. 14. f14:**
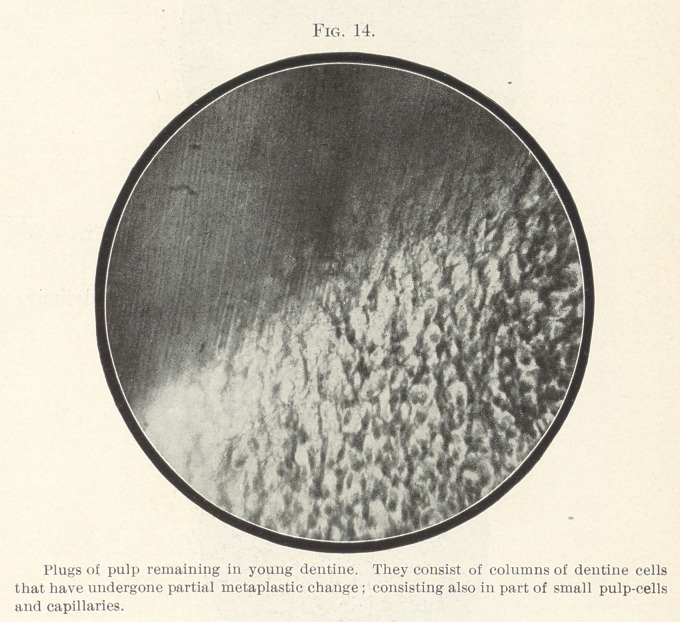


**Fig. 15. f15:**
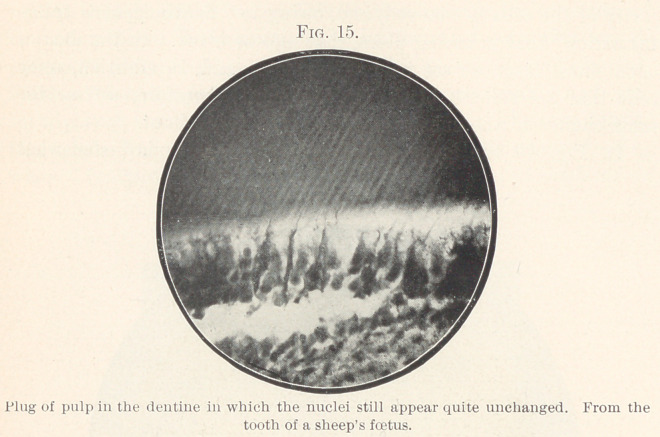


**Fig. 16. f16:**
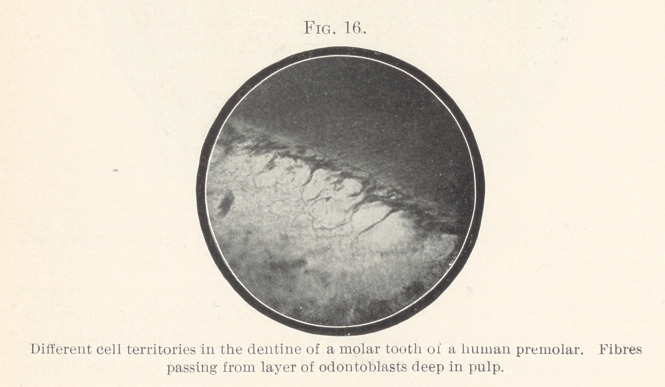


**Fig. 17. f17:**
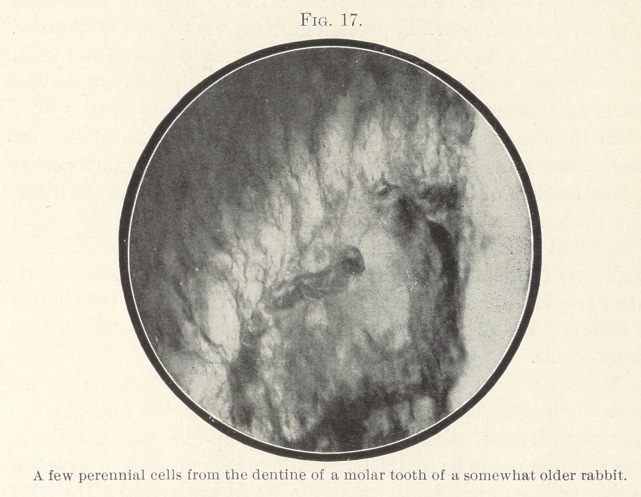


**Fig. 18. f18:**
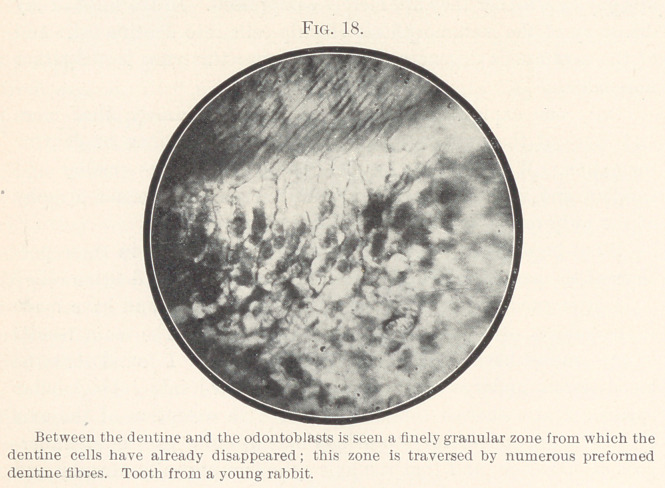


**Fig. 19. f19:**
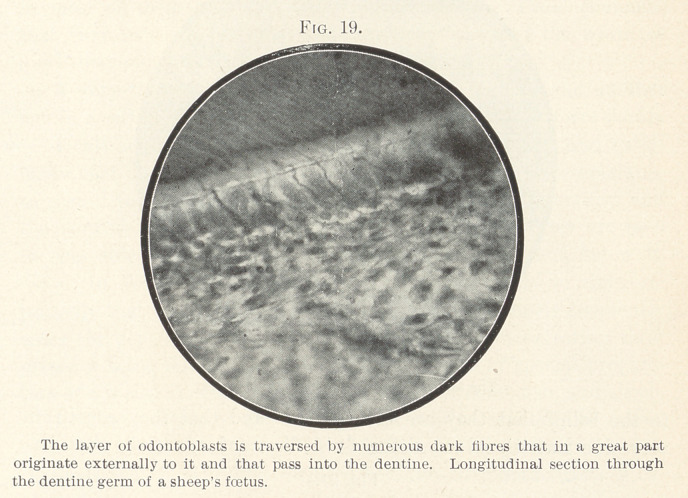


**Fig. 20. f20:**
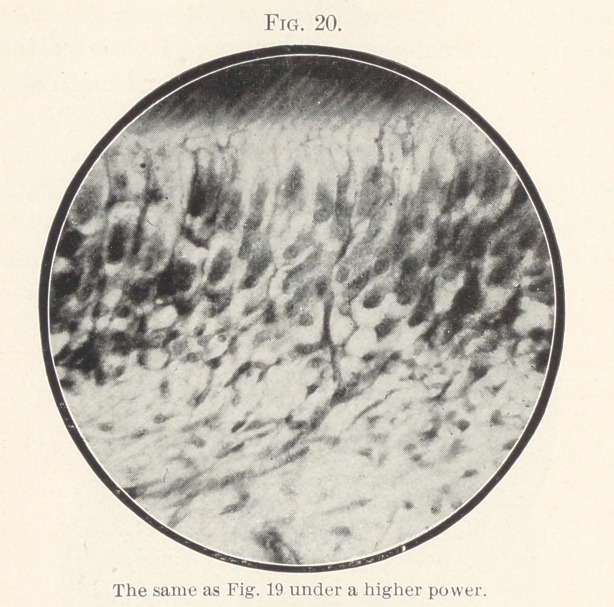


**Fig. 21. f21:**